# Synthesis of Poly (2-Acrylamido-2-methylpropanesulfnoinc Salt) Modified Carbon Spheres

**DOI:** 10.3390/polym15173510

**Published:** 2023-08-23

**Authors:** Na Su

**Affiliations:** 1 Department of Printing and Packaging Engineering, Shanghai Publishing and Printing College, Shanghai 200093, China; suna@whu.edu.cn; 2 School of Materials and Chemistry, University of Shanghai for Science and Technology, Shanghai 200125, China

**Keywords:** carbon spheres, anionic spherical brushes, surface-initiated polymerization, NaAMPS

## Abstract

The paper reports a facile synthesis of novel anionic spherical polymer brushes which was based on grafting sodium 2-acrylamido-2-methylpropane-1-sulfonate from the surface of 4,4′-Azobis (4-cyanopentanoyl chloride)-modified carbon spheres. Various characterization methods involving a scanning electron microscope, energy dispersive X-ray spectroscopy, Fourier transform infrared spectrum, and thermo-gravimetric analysis were utilized to analyze the morphology, chemical composition, bonding structure, and thermal stability, respectively. The molecular weight (*Mw*) and polydispersity (*Mw*/*Mn*) of brushes were 616,000 g/mol and 1.72 determined by gel permeation chromatography experiments. Moreover, the dispersibility of ASPB in water and in the presence of aqueous NaCl solutions of different concentrations was investigated. Results show that the dispersibility of carbon spheres has been enhanced owing to grafted polyelectrolyte chains, while the zeta potential of the particle decreases and its brush layer shrinks upon exposure to sodium ions (Na^+^).

## 1. Introduction

In recent years, the preparation of carbon spheres by the hydrothermal reaction has attracted extensive attention from researchers in science and industry. With the advantages of sustainability, environmental friendliness, low cost, and the ease of synthesis, hydrothermal carbon spheres have potential applications in the fields of catalysts [1,2], adsorbents [3,4], and lithium batteries [5,6]. Consequently, the chemical modification of hydrothermal carbon spheres is favorable to its application.

Generally, two chemical modification techniques of “in-situ modification” [7] or “post-modification” [8,9] are commonly used. The “in-situ modification” method may be subjected to the negative effect introduced by the hydrothermal carbonization reaction [10], while the “post-modification” technique is based on the synthesized carbon spheres by one-step hydrothermal reaction of nitrogen-containing groups. The reaction conditions and the type and nature of functional groups can be easily controlled. [11]. So, efforts to explore green, simple, and efficient modification methods have led to numerous studies on the preparation [12,13] and application [14] of functionalized hydrothermal carbon spheres.

Polyelectrolytes refer to macromolecules with ionizable groups which are in polar solvents, making the polymer chains charged. As an important soft substance, polyelectrolytes have important effects on the structure, stability, and interaction of molecular assemblies. The modification of carbon materials, such as carbon nanotubes [15], graphene [16], etc., using polyelectrolytes has become a research hotspot in the field of materials science. If the polyelectrolytes are densely grafted to the surface of carbon spheres to form a brush-like conformation, spherical polyelectrolyte brushes (SPB) result [17]. Recently, our previous works have shown that SPB plays an essential role in the synthesis of conductive nanocomposites for application in printed electronics due to its brush-like conformation [18,19]. However, there are few reports on the hydrothermal modification of carbon spheres by using poly (sodium 2-acrylamido-2-methylpropane-1-sulfonate) which has been widely used in supercapacitors [20] and drug release [21]. 

**Figure 1:**
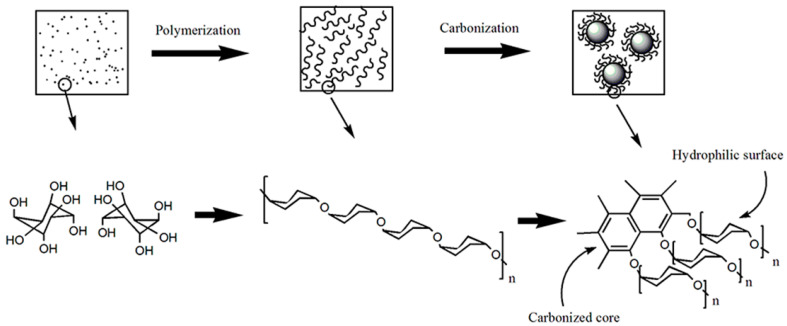
Preparation of CS by hydrothermal method.

**Figure 2:**
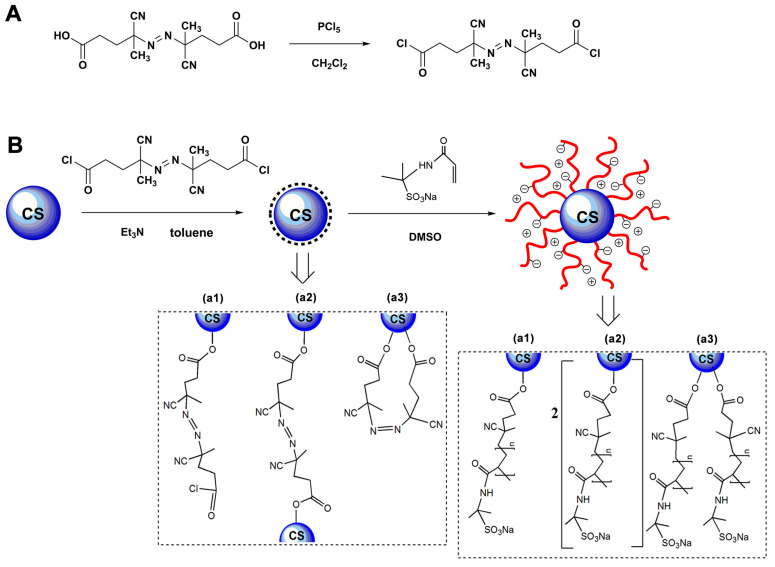
Schematic representations of synthesis process of ASPB. (**A**) Synthesis of 4,4’-Azobis(4-cyanopentanoyl chloride), (**B**) Immobilization of azo initiator and synthesis of ASPB. (**a1**) Single-ended form grafted on CS, (**a2**) double-ended form grafted on hetero-CS, and (**a3**) double-ended form grafted on homo-CS.

In this paper, novel anionic spherical polyelectrolyte brushes (ASPB) consisting of a carbon spheres (CS) core and a well-defined shell of poly (sodium 2-acrylamido-2-methylpropane-1-sulfonate) (PAMPSNa) brushes have been successfully synthesized by surface-initiated polymerization (“grafting from”). CS is prepared by a hydrothermal method and its synthesis process is described in Figure 1. The surface-initiated polymerization of sodium 4-vinylbenzenesulfonate monomer, initiated by the azo initiator introduced onto the surface of CS is ultimately carried out (see Figure 2). This design process not only allows for the controllable molecular structure of nanocomposites, but also maintains the intrinsic chemical structure characteristics of polymer. In addition, this approach is simple, practical, and maneuverable.

## 2. Materials and Methods

### 2.1. Materials

Glucose (Sinopharm Chemical Reagent Co., Ltd., Shanghai, China); Sodium 2-acrylamido-2-methylpropane-1-sulfonate (NaAMPS) and 4,4′-azobis(4-cyanovaleric acid) (ACVA) (Aladdin Reagent Co., Ltd., Shanghai, China); phosporus pentachloride (PCl_5_, Shanghai Tingxin Chemical Factory, Shanghai, China); dichloromethane, triethylamine; and dimethyl sulfoxide (DMSO) are all purchased from Guoyao Group of Chemical Reagents Ltd., Shanghai, China. Other chemicals and solvents including toluene, methanol, and ethanol are of an analytical grade and used without any further purification.

### 2.2. Preparation of Anionic Spherical Polyelectrolyte Brushes

As shown in Figure 1, the carbon spheres (CS) were prepared by a glucose hydrothermal method [22]. 

The reaction protocol in Figure 2 clearly showed the sequence used to synthesize the azo initiator and spherical brushes. Firstly, the synthesis process of 4,4′-Azobis (4-cyanopentanoyl chloride) was introduced. A total of 2.8 g of 4,4′-azobis(4-cyanovaleric acid) was dispersed in 50 mL dry dichloromethane. The suspension was cooled to 0 °C and stirred for 30 min under a nitrogen atmosphere. Then, 4.0 g of PCl_5_ was added dropwise to the above mixture. After several hours, the reaction mixture was filtered to remove unreacted phosphorus pentachloride. The remaining solution was concentrated to about 20 mL. A white solid was obtained after a solution of 200 mL old n-hexane was added and then filtered and dried under vacuum overnight.

Secondly, the immobilization routes for azo initiator on the surface of carbon spheres were preformed 0.1 g of CS was dispersed in 3 mL dry toluene with ultrasonic treatment for 10 min. Subsequently, 0.32 g of 4,4′-azobis(4-cyanopentanoyl chloride) and 0.3 mL triethylamine were added. The reaction was carried out at room temperature for 24 h. The products were collected by centrifugation and washed thoroughly by several sonication and centrifugation cycles using ethanol and deionized water before drying overnight in a vacuum oven.

Finally, anionic spherical polyelectrolyte brushes were synthesized based on the above-mentioned processes. Azo initiator-immobilized CS and 3.3 g of sodium 4-vinylbenzenesulfonate monomer were added to 20 mL DMSO, followed by polymerization at 60 °C for 6 h under a flow of nitrogen. The resulting products were purified and vacuum-dried at 60 °C for 12 h.

### 2.3. Characterization

Qualitative structure analysis based on peak intensities was performed by the Fourier transform infrared spectrum using a Fourier transform infrared spectrometer (Nicolet AVATAR 360FT, Madison, WI, USA). X-ray photoelectron spectroscopy (XPS) measurements were carried out on an FEI “Quanta 200” instrument operating at a voltage of 30 kV with MnKα radiation. The morphologies of samples were observed using a high-resolution Quanta 200 scanning electron microscope operated at 30 kV. The chemical composition of ASPB was examined by energy-dispersive X-ray diffraction spectroscopy. With quantitative element analysis by EDX test, it was possible to calculate the graft density of the initiator on the surface of CS according to the following Formula (1).

(1) $$\delta =\frac{g_{E}}{M_{E}Z}$$

where *δ* is the graft density, *g_E_* is the percentage content of C, H, and N elements, respectively, *M_E_* represents the molecular weight of the corresponding element, and *Z* stands for the number of the corresponding element.

**Figure 3:**
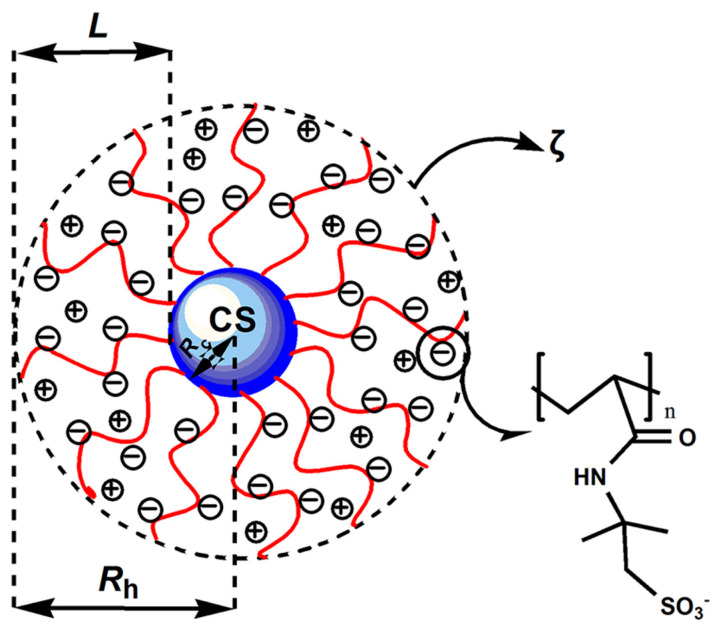
A sketch of ASPB in this study.

The hydrodynamic radius R_h_ of ASPB and zeta potential were tested by Zeta Potential/Particle Sizer (ZLS) (Nicomp 380, Carolina, PR, USA). Figure 3 demonstrated this model system termed ASPB. The core radius R_c_ denoted azo initiator-immobilized CS, from which polyelectrolyte chains (PAMPS) were grafted. L represented the thickness of the brush layer, R_h_ the hydrodynamic radius and ζ the zeta potential.

The molecular weight distribution of brushes was investigated by gel permeation chromatography (GPC) (Spectra SERIES P100). The thermal stability of the samples was explored by thermo-gravimetric analysis (TGA) using a SETSYS-1750[SETARAM] instrument with heating to 650 °C at 10 °C/min in an N_2_ atmosphere. A conductivity meter (DDS-12DW Microprocessor Conductivity Meter, A&E Laboratory Co., Ltd., Guangzhou, China) was used to determine the conductive performance of samples. 

## 3. Results and Discussion

### 3.1. FTIR and XPS Analyses

**Figure 4:**
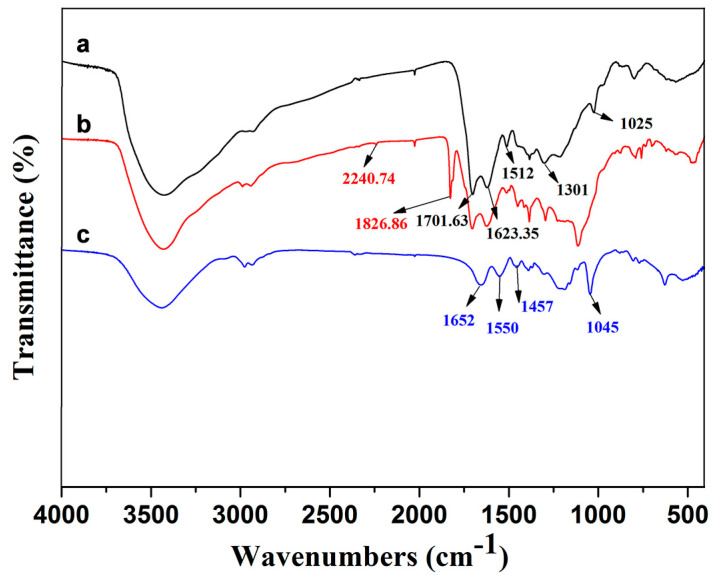
FTIR spectra of (a) CS, (b) azo initiator-immobilized CS, and (c) ASPB.

The examination of the infrared spectrum of CS, azo initiator-immobilized carbon spheres, and ASPB shown in Figure 4 provides their chemical bond structure. For CS, the assignment of the peaks at 1701.63 cm^−1^ and 1623.35 cm^−1^ corresponds to the C=O and C=C vibrations, respectively. In addition, the appearance of the C-OH stretching and -OH bending vibrations in the region of 1025~1512 cm^−1^ (Figure 4a) confirms the presence of large numbers of residual hydroxy groups. In the infrared spectrum of initiator-immobilized CS (Figure 4b), the absorptions peak at 2240.74 and 1826.86 cm^−1^ are usually representative of the -CN stretching vibrations and C=O stretching vibration, respectively. It reveals that the surface of CS has been attached successfully by azo initiator. The spectrum of ASPB (Figure 4c) displays the bending vibration absorption peak at 1652 cm^−1^ of C=O (-CONH_2_) and the symmetric SO_3_ stretching band at 1045 cm^−1^, proving the presence of grafted NaAMPS chains [23].

**Figure 5:**
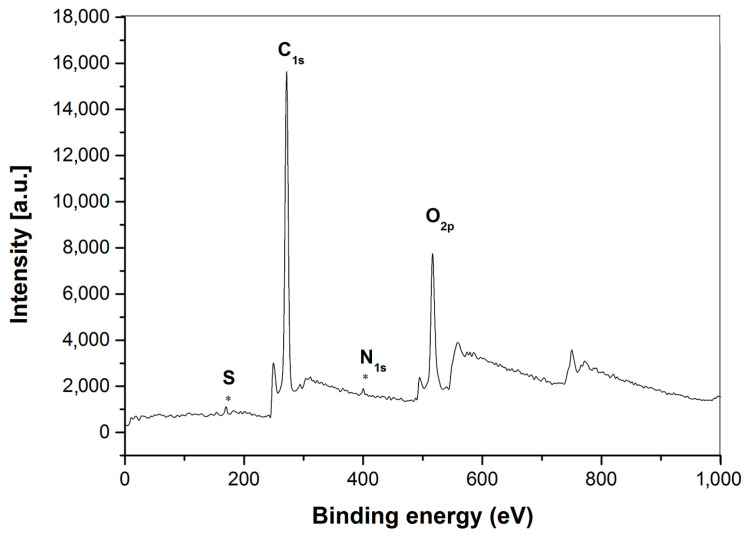
XPS spectrum of wide-region spectroscopy of ASPB.

Figure 5 shows typical XPS wide-scan spectra for ASPB. The nitrogen signals and sulfur are observed in the survey spectrum of ASPB, which is in accordance with the FTIR studies.

### 3.2. Morphologies and EDX Analyses

**Figure 6:**
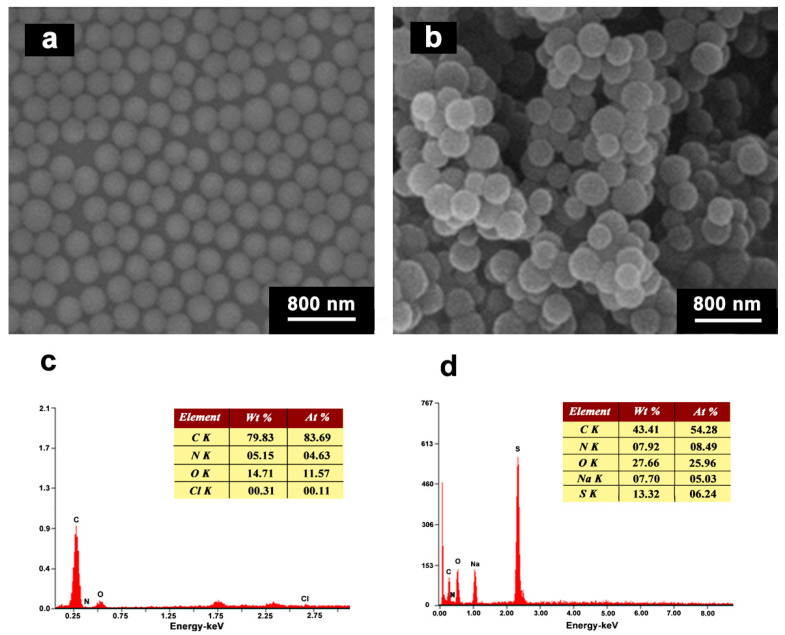
SEM images of (**a**) CS and (**b**) ASPB; EDX analyses of (**c**) azo initiator-immobilized CS and (**d**) ASPB.

The morphologies of CS, and ASPB are clearly demonstrated by SEM images. A smooth surface (Figure 6a) is shown in SEM images of CS, and the radius *R* of CS is ca. 270 nm determined by DLS experiments. The morphologies of ASPB are dissimilar from those of CS; fuzzy edges can be observed in their SEM (Figure 6b) images, proving the successful preparation of ASPB. The hydrodynamic radius *R*_h_ of ASPB is ca. 425 nm according to the DLS result.

The EDX analyses represent the chemical composition of azo initiator-immobilized CS (see Figure 6c) and ASPB (see Figure 6d). As observed in Figure 6c, the signals corresponding to N and Cl appear on the spectrum, indicating that the immobilization of the azo initiator on the CS surface is achieved. The EDX analysis of ASPB shown in Figure 6d obviously contains C, N, O, Na, and S elements, and their contents are 43.41%, 7.92%, 27.66%, 7.70%, and 13.32%, respectively. Compared with the azo initiator-immobilized CS, two new elements (S and Na) contained in polymerized monomers appear, which further supports the polymerization reaction. In addition, the graft density of the azo initiator on the surface of carbon spheres is 919.6 mmol/g according to Formula (1).

### 3.3. Molecular Weight

**Figure 7:**
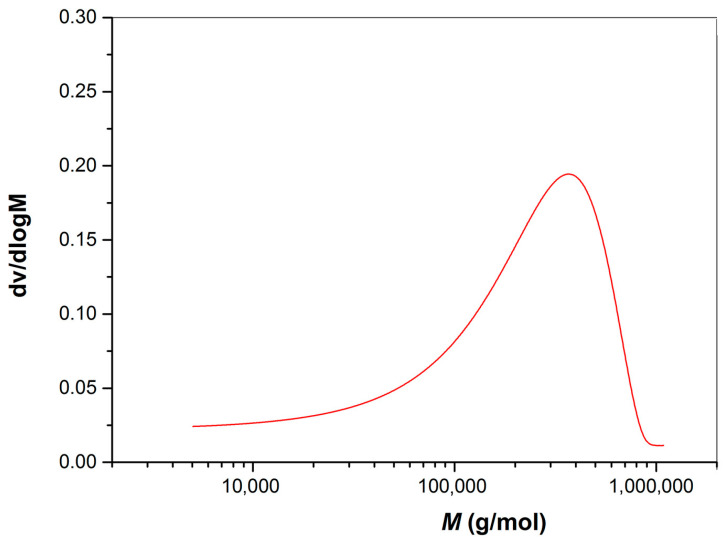
GPC curves of PAMPS chains.

According to previous studies [24], brushes and free polymers are much alike in molecular weight and their distribution. Hence, the molecular weight of brushes is determined after collecting and purifying the free polymers in the solution. Taking 0.1 M NaCl (aq) as the mobile phase, PEG is used as the internal standard at room temperature. The GPC measurements disclose that Mw is 616,000 g/mol, and polydispersity Mw/Mn is 1.72 (see Figure 7).

### 3.4. TGA Curves

**Figure 8:**
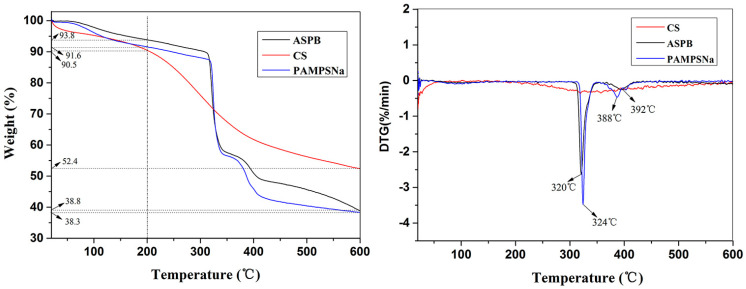
TG-DGT curves of CS, PAMPSNa, and ASPB.

Figure 8 represents the TG-DTG curves of CS, PAMPSNa, and ASPB. All the samples display weight loss below 200 °C, which is due to the removal of absorbed water. For CS, the total weight loss is 39.2 wt % in the time range of 200–600 °C, and the weight loss between 200 °C and 400 °C is mainly related to the loss of CS [25]. Although the TGA curves of ASPB and PAMPSNa exhibit similarity in shape, differences are observed in DTG peaks. For pure PAMPSNa, two endothermic peaks at 324 °C and 388 °C appear, which is attributed to the decomposition of PAMPSNa with a weight loss of 53.3%. It is mainly because of the degradation of the polymer backbone [26]. In the case of ASPB, the endothermic peaks from 300 °C to 400 °C are also observed (320 °C and 392 °C), proving that the PAMPSNa has been bonded to CS.

### 3.5. Solution Conductivity

**Figure 9:**
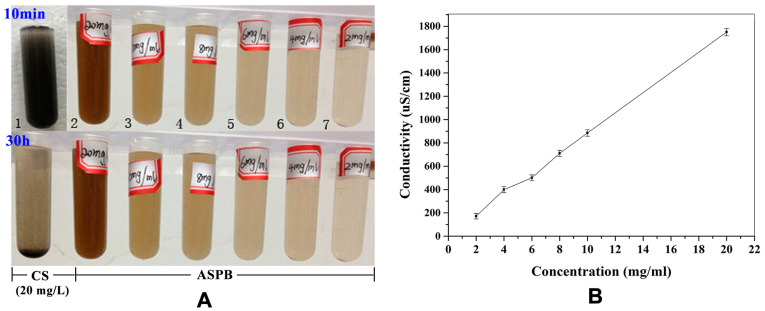
(**A**) Qualitative and (**B**) quantitative analysis of solution dispersibility of CS (20 mg/L) and ASPB at different concentrations.

Figure 9 shows the dispersibility of ASPB in water, which is reflected by the conductivity of the solution at different concentrations. As demonstrated by Figure 9A, the CS (20 mg/L, No. 1) and ASPB (No. 2~7) with different concentrations are firstly dispersed in water under ultrasonic dispersion for 10 min and then left for 30 h. We can clearly see that CS and ASPB are found to be uniformly dispersed in the aqueous solution at first. However, 30 h later, CS settles to the bottom of the centrifuge tube, while ASPB are uniformly dispersed and stable in the aqueous solution. The reason for this may be that the agglomeration of CS is prevented by the flexible long chains of PAMPSNa, which makes the particles well dispersed in water. Undoubtedly, the entry of PAMPSNa chains can improve the hydrophilicity of ASPB.

As a quantitative analysis method, the solution conductivity of samples at different concentrations is tested (see Figure 9B). After testing the conductivity of 20 mg/mL aqueous solution of CS (3.68 μS/cm), the conductivities of the aqueous solutions of ASPB with concentrations of 2, 4, 6, 8, 10, and 20 mg/mL are 171.9 μS/cm, 399 μS/cm, 501 μS/cm, 710.7 μS/cm, 885 μS/cm, and 1751 μS/cm, respectively. It can be seen from the figure that with the increase in polyelectrolyte concentration, the conductivity of the solution also increases significantly and changes almost linearly. This means that the polymer chains on the surface of CS have the characteristics of high charge density and high molecular weight, which is consistent with the characteristics of free radical polymerization.

### 3.6. Zeta Potential and R_h_

**Figure 10:**
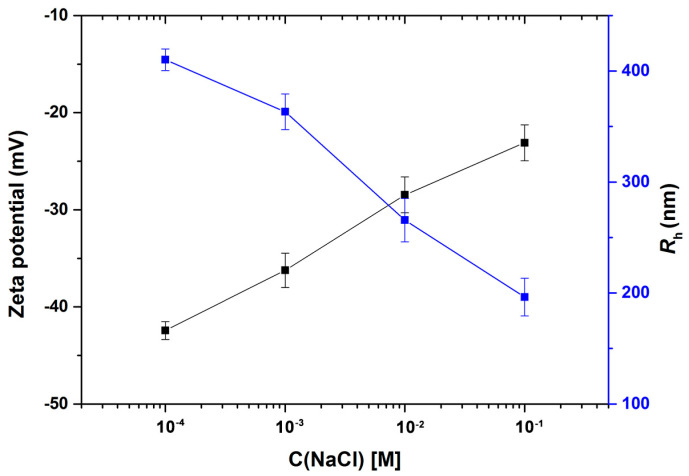
Zeta potential and *R*_h_ of ASPB as a function of aqueous NaCl solutions of different concentrations (T = 25 °C, pH = 6, c(ASPB) = 1 mg/mL).

Figure 10 demonstrates the *R*_h_ of ASPB and zeta potential as a function of aqueous NaCl solutions of different concentrations. It is observed that the reduction in the values of zeta potential and *R*_h_ occurs with increasing salt concentration. At the low salt concentration, the strong steric repulsions between grafted polyelectrolyte chains make the brush layer of ASPB form an extended conformation, thus obtaining a stable dispersion system. Raising the ionic strength by adding salt concentration, however, will diminish hydrodynamic radius. A shrinking of the brush layer becomes pronounced when the salt concentration is around 0.1 M, which means that electrostatic interaction plays no role anymore. This effect is very accurately captured by the theory of Hariharan et al. [27]. Meanwhile, the dispersion of ASPB is no longer stable. The reason for this is presumably that the screen electrostatic interaction prevails with the increase of the ionic strength, resulting in a continuous collapse of the brush layer. There is hardly any stretching of the polyelectrolyte chains, which has the effect of moving the shear plane closer to the surface, thus decreasing the zeta potential of particles [28]. 

## 4. Conclusions

In this study, novel ASPB have been successfully synthesized by grafting anionic polyelectrolyte chains on the surface of carbon spheres using radical polymerization technique. Synthesized ASPB displays uniform spherical morphology, long polymer chains, and narrow molecular weight distribution. The dispersibility of ASPB in water is explored by conductivity tests. Results indicate that the solution’s conductivity of ASPB with the concentration of 20 mg/mL is 476 times the carbon spheres at the same concentration. Grafted polyelectrolyte chains improve the dispersibility of carbon spheres. However, a decrease in the zeta potential of the particle and a shrinking of its brush layer upon exposure to sodium ions (Na^+^) is observed.

## Data Availability

All data generated or analyzed are included as figures in this published paper.

## References

[1] Ballauff M. (2007). Spherical polyelectrolyte brushes. Prog. Polym. Sci..

[2] Yang Q., Li L., Zhao F., Wang Y., Ye Z., Guo X. (2019). Generation of MnO_2_ nanozyme in spherical polyelectrolyte brush for colorimetric detection of glutathione. Mater. Lett..

[3] Lu Y., Ballauff M. (2016). Spherical polyelectrolyte brushes as nanoreactors for the generation of metallic and oxidic nanoparticles: Synthesis and application in catalysis. Prog. Polym. Sci..

[4] Hasani S., Mohamadnia Z., Kazem F. (2021). Preparation of microbeads grafted with poly (2-(acryloyloxy)ethyl]trimethylammonium chloride) cationic polyelectrolyte as recyclable and effective adsorbents for organic dyes. React. Funct. Polym..

[5] Suchithra P.S., Vazhayal L., Mohamed A.P., Ananthakumar S. (2012). Mesoporous organic-inorganic hybrid aerogels through ultrasonic assisted sol–gel intercalation of silica-PEG in bentonite for effective removal of dyes, volatile organic pollutants and petroleum products from aqueous solution. Chem. Eng. J..

[6] Wang W.H., Li L., Henzler K., Lu Y., Wang J.Y., Han H., Tian Y., Wang Y., Zhou Z., Lotze G. (2017). Protein Immobilization onto Cationic Spherical Polyelectrolyte Brushes Studied by Small Angle X-ray Scattering. Biomacromolecules.

[7] Han L., Yan B., Zhang L., Wu M., Wang J., Huang J., Deng Y., Zeng H. (2018). Tuning protein adsorption on charged polyelectrolyte brushes *via* salinity adjustment. Colloid Surf. A.

[8] Zhang X.Z., Huang Y., Fu K.Q., Yuan S.J., Huang C., Li H.B. (2016). Preparation and performance of cationic flocculant for papermaking based on the graft polymerization of cationic chains from colloidal silica particles. Colloid Surf. A.

[9] Mei Y., Abetz C., Birkert O., Schȁdler V., Leyrer R.J., Ballauff M. (2006). Interaction of Spherical Polyelectrolyte Brushes with Calcium Carbonate and Cellulose Fibers: Mechanistic Studies and Their Application in Papermaking. J. Appl. Polym. Sci..

[10] Su N. (2015). Polyaniline-Doped Spherical Polyelectrolyte Brush Nanocomposites with Enhanced Electrical Conductivity, Thermal Stability, and Solubility Property. Polymers.

[11] Su N. (2015). Improving Electrical Conductivity, Thermal Stability, and Solubility of Polyaniline-Polypyrrole Nanocomposite by Doping with Anionic Spherical Polyelectrolyte Brushes. Nanoscale Res. Lett..

[12] Kobayashi M., Yamaguchi H., Terayama Y., Wang Z., Ishihara K., Hino M., Takahara A. (2009). Structure and surface properties of high-density polyelectrolyte brushes at the interface of aqueous solution. Macromol. Symp..

[13] Xiang L., Qiu M., Shang M.J., Su Y.H. (2021). Continuous synthesis of star polymers with RAFT polymerization in cascade microreactor systems. Polymer.

[14] Riley J.K., Matyjaszewski K., Tilton R.D. (2018). Friction and adhesion control between adsorbed layers of polyelectrolyte brush-grafted nanoparticles via pH-triggered bridging interactions. J. Colloid Interf. Sci..

[15] Slowikowska M., Wojcik A.J., Wolski K., Hatalak A., Zapotoczny S. (2021). Light-promoted synthesis of surface-grafted polymers bearing pyridine groups by metal-free ATRP in microliter volumes. Polymer.

[16] Gao T., Nie M., Luo J., Huang Z., Sun H., Guo P., Xue Z., Liao J., Li Q., Teng L. (2021). Nickel sulfides supported by carbon spheres as efficient catalysts for hydrogen evolution reaction. Electrochem. Commun..

[17] Norouzi Z., Mahmoudi Najafi S.H., Mozaffari S.A. (2022). Silver-loaded carbon sphere-in-rod 3D nano-architectures as electrode material for supercapacitors. Diam. Relat. Mater..

[18] Zhang C., Ren X., Kou L., Zhang X., Wang R., Xie L., Fan C. (2021). Facile synthesis of nitrogen-rich porous carbon spheres assisted by NaNH_2_ as a bifunctional activator and nitrogen source for CO_2_ capture. J. Environ. Chem. Eng..

[19] Chen X., Wu Y., Gu W., Zhou M., Tang S., Cao J., Zou Z., Ji G. (2022). Research progress on nanostructure design and composition regulation of carbon spheres for the microwave absorption. Carbon.

[20] Afrifah V.A., Phiri I., Hamenu L., Madzvamuse A., Lee K.S., Ko J.M. (2020). Electrochemical properties of poly (2-acrylamido-2-methylpropane sulfonic acid) polyelectrolyte containing zwitterionic silica sulfobetaine for supercapacitors. J. Power Sources.

[21] Ashames A., Pervaiz F., Al-Tabakha M., Khalid K., Hassan N., Shoukat H., Buabeid M., Murtaza G. (2022). Synthesis of cross-linked carboxymethyl cellulose and poly (2-acrylamido-2-methylpropane sulfonic acid) hydrogel for sustained drug release optimized by Box-Behnken Design. J. Saudi Chem. Soc..

[22] Mkhari O., Ntuli T.D., Coville N.J., Nxumalo E.N., Maubane-Nkadimeng M.S. (2021). A comparison of fluorescent N-doped carbon dots supported on the surface of hollow and solid carbon spheres, and solid silica spheres. Diam. Relat. Mater..

[23] Su N., Li H.B., Hang Y., Zhang X.Z. (2015). Synthesis of Salt Responsive Spherical Polymer Brushes. J. Nanomater..

[24] Nikolaou V., Simula A., Droesbeke M., Risangud N., Anastasaki A., Kempe K., Wilson P., Haddleton D.M. (2016). Polymerisation of 2-acrylamido-2-methylpropane sulfonic acid sodium salt (NaAMPS) and acryloyl phosphatidylcholine (APC) *via* aqueous Cu (0)-mediated radical polymerization. Polym. Chem..

[25] Zhang H., Rȕhe J. (2005). Swelling of poly (methacrylic acid) brushes: Influence of monovalent salts in the environment. Macromolecules.

[26] Asifjaved H.M., Ahmad M.I., Que W.X., Qureshi A.A., Sarfaraz M., Hussain S., Lqbal M.Z., Nisar M.Z., Shahid M., Algarni T.S. (2021). Encapsulation of TiO_2_ nanotubes with Cs nanoparticles to enhance electron injection and thermal stability of perovskite solar cells. Surf. Interfaces.

[27] Hariharan R., Biver C., Russel W.B. (1998). Ionic strength effects in polyelectrolyte brushes: The counterion correction. Macromolecules.

[28] Kao J.Y., Cheng W.T. (2020). Study on Dispersion of TiO_2_ Nanopowder in Aqueous Solution via Near Supercritical Fluids. ACS Omega.

